# Supplementary material for: MetaRibo-Seq measures translation in microbiomes
Source: Nat Commun. 2020 Jun 29;11:3268. doi: 10.1038/s41467-020-17081-z (PMC7324362; doi:10.1038/s41467-020-17081-z)
Supplement: Supplementary file 10 — Supplementary Data 7 [file 41467_2020_17081_MOESM10_ESM.zip › File2/Confidence_High_Taxonomy/291294_out.krona.html]

Javascript must be enabled to view this page.

members
magnitude
magnitudeUnassigned
count
unassigned
taxon
rank

291294\_out

34

2
34
superkingdom

34
phylum
976

200643
class
34

order
34
171549

34
family
815

816
genus
34

species
27

SRS011405\_contig\_number\_1480SRS013158\_contig\_number\_4835SRS016753\_contig\_number\_1435SRS017247\_contig\_number\_312SRS017859\_contig\_number\_1115SRS019286\_contig\_number\_contig-100\_517.45794SRS023914\_contig\_number\_17912SRS023971\_contig\_number\_1069SRS024009\_contig\_number\_2931SRS024075\_contig\_number\_5045SRS024549\_contig\_number\_1466SRS042284\_contig\_number\_4349SRS043841\_contig\_number\_2266SRS044535\_contig\_number\_13886SRS045739\_contig\_number\_1302SRS046068\_contig\_number\_3988SRS052027\_contig\_number\_1810SRS063813\_contig\_number\_1335SRS074670\_contig\_number\_1626SRS077454\_contig\_number\_15455SRS104636\_contig\_number\_30952SRS104912\_contig\_number\_8020SRS143181\_contig\_number\_contig-100\_206.77542SRS147139\_contig\_number\_3047SRS147271\_contig\_number\_23787SRS147977\_contig\_number\_624SRS149075\_contig\_number\_contig-100\_329.109981
371601


SRS015486\_contig\_number\_431SRS046369\_contig\_number\_1644SRS076756\_contig\_number\_42SRS1041130\_contig\_number\_930SRS143466\_contig\_number\_30882SRS893285\_contig\_number\_655
6
species
818

871324
1

SRS020233\_contig\_number\_12024
species
